# A novel pressed coal from citrus and cooking oil wastes using fungi

**DOI:** 10.1186/s40643-022-00582-8

**Published:** 2022-09-05

**Authors:** Mohamed S. Hasanin, Amr H. Hashem, Hassan M. Abu Hashish, Mohamed Abdelraof

**Affiliations:** 1 Cellulose & Paper Department, National Research Centre, 33 El Bohouth St., Dokki, P.O. 12622, Giza, Egypt; 2 Botany and Microbiology Department, Faculty of Science, Al-Azhar University, Cairo, 11884 Egypt; 3 Mechanical Engineering Department, Engineering Research Division, National Research Centre, Giza, Egypt; 4 Microbial Chemistry Department, National Research Centre, Dokki, Cairo, 12622 Egypt

**Keywords:** Cooking oil waste, Screw press, Citrus trees fibers, Fuels, *Aspergillus flavus*

## Abstract

Nowadays renewable energy with low prices is a global target that has taken the attention to compare alternatives energy sources with fossil fuels. Therefore, this study was established to find suitable and sustainable alternative low-cost fuels source. Cooking oil waste (COW) was mixed with non-pretreated citrus tree fibers (CTF) (0.5 mL to 1 g ratio) and pressed to formulate coal (CTF/COW). Otherwise, this mixture was subjected to in situ fungal pretreated using *Aspergillus flavus* isolate to simplify the mixture composition and pressed to offer in a usable form with enhancing their heating value for the first time. CTF/COW was characterized using attenuated total reflection Fourier-transform infrared spectroscopy (ATR-FTIR), scanning electron microscope (SEM) and thermal analysis (TGA) before and after treatment. The fungal isolate was observed with enzyme productivity and activity of CMCase, avicelase, xylanase, mannanase, α-glucosidase, β-glucosidase, lignin peroxidase and lipase according to enzyme assays and the chemical compositions of CTF before and after fungal treatment, where the best PH for enzymes extraction was between 5 and 7. The fungal enzymes increased the heating value by about two and half folds in comparison with non-pretreated coal. Moreover, the calorific value of tCTF/COW was 43,422 kJ/kg, which was higher than CTF recorded 18,214 kJ/kg and COW recorded 39,823 kJ/kg. Our result suggests that fungal treatment of the mixture of citrus trees and cooking oil waste presents as a promising low-cost and eco-friendly coal.

**Graphical Abstract:**

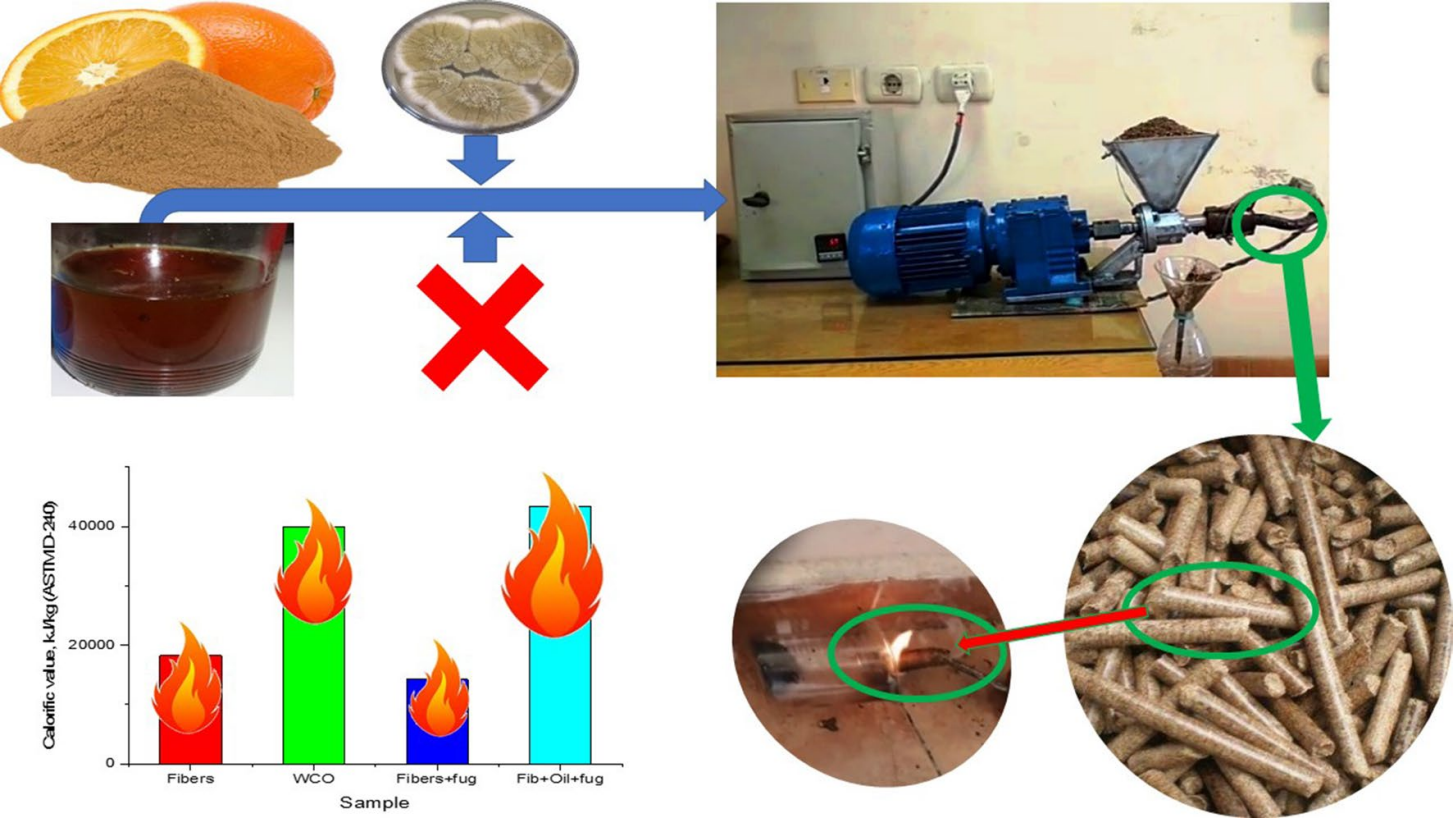

## Introduction

The renewable energy fuels based low cost and sustainable materials is one of the most attractive critical issues overall the earth (Ali et al. 2018; Hashem et al. 2019). The environmental hazard of energy generation from fossil fuels became more indiscriminate and instability, this leads to searching for finding new alternative energy sources that have a less-pollutant as well as low cost (Majid and Society 2020). In this regard, lignocellulosic wastes have a vast potential the alternative green fuel sources, and value-added bioproducts (FitzPatrick et al. 2010). In this context, biotechnology approaches add a great value to waste modification treatment, especially those using enzymatic treatments (Monteiro et al. 2021; Moreira et al. 2020). Enzymatic modification of lignocellulosic biomass usually corresponds to the lignin, hemicelluloses, and cellulose (Hasanin et al. 2018; Menon et al. 2012; Tawfik et al. 2021). Fungi that have lignocellulolytic ability usually secret extracellular enzymes, such as cellulases, hemicellulases, peroxidases, and laccase (Kantharaj et al. 2017). Among the largest lignocellulosic raw materials, citrus tree fibers (CTF) are portrayed as zero-value biomass, which can use for environmental and economical issues as well. Indeed, the increase of planting areas and the accumulation of this residue into the environment that prerequisite strategies to handle and reduce (Rahman et al. 2022). In this way, conversion of the CTF by fungal enzymes degradation under solid-state fermentation is considered as a novel green eco-friendly treatment strategy for production a renewable, sustainable cost-effective coal with high heating value (Tang et al. 2022). On the other hand, COW is a popular domestic waste generated over all the world as a waste of vegetable oil after using in the cooking and frying of food (De Feo et al. 2020). COW refers mainly to frying oil used at high temperatures, edible fat mixed in kitchen waste and oily wastewater directly discharged into the environment (Zhang et al. 2012). Unfortunately, the recovery of this type of waste has not restricted collection planning in many countries that could be considered solid or liquid waste (Ortner et al. 2016). In addition, screw press is a good selection to produce a pressed high-density coal. This method was offered a simple and effective eco-friendly non-hazardous technique to formulate a coal material with unique characteristics in comparison with other convention coal. Therefore, in this work the new promising formula of fungal treated biowastes included citrus trees fibers and cooking oil waste this followed by screw press to produced renewable, sustainable cost-effective coal with high heating value.

## Materials and methods

### Wastes collection

Citrus trees fibers was obtained from citrus trees trimmings, since it were collected from some local farms located in Giza, Egypt. The cooking oil waste was collected from local fast-food restaurant in Egypt.

### Chemical reagents

p-Nitrophenyl b-D-glucopyranoside, p-nitrophenyl α-glucopyranoside, locust bean gum, and beech wood xylan were purchased from Sigma-Aldrich Chemical Co. (St. Louis, MO, USA). Carboxymethylcellulose (CMC), microcrystalline cellulose (MCC) were purchased from Merck (Germany). All other reagents used were of the highest grade available.

### Fungal strains and culture preservation conditions

Six fungal strains were used in this study, *Rhizopus oryzae* (accession number MG518370), *Rhizopus microsporus* (accession number MK623262), *Aspergillus niger* RCMB 02724*, A. fumigatus* RCMB 02568*, A.* flavus EGYPTA5 and *A. terreus* RCMB 02574. Fungal strains were cultured on Malt Extract Media (MEA) and incubated at 28 ± 2 °C for 3–4 days, then kept at 4 °C for further use (Fouda et al. 2015; Hasanin and Hashem 2020; Khalil et al. 2019; Mohamed Aly Khalil and Hosny Hashem 2018).

### CTF preparation

CTF was pretreated by grinding in meshes about 2 mm in dimeter and 0.5–3 cm in length. Afterword, the CTF washed with distilled water and dried at 60º C for 18 h.

### Solid-state fermentation (SSF)

SSF medium was prepared as follows: 20 g of dried pretreated CTF was put in a 500-mL flask and autoclaved for 15 min at 121ºC. After cooling, pretreated CTF was moistened uniformly with sterile distilled water under septic condition at 70% to produce tCTF. In the case of pretreated CTF with COW, the same method used plus 10 mL of COW. These media were inoculated with a known number of spores (1.5X10^6^ /mL) individually, then incubated for 2 weeks at 28 ± 2 °C.

### Enzyme assay

The activity of CMCase was assayed based on the method of (Mandels et al. 1974). Briefly, appropriately diluted supernatant and 0.5 mL of carboxymethyl cellulose (CMC, 2% w/v) in citrate buffer (50 mM, pH 4.8) were mixed in equal volumes, and the enzyme reaction mixture was incubated at 50 °C for 30 min. Avicelase activity was determined under similar conditions, with the exception that the enzyme reaction proceeded for 2 h in 1.0 mL of acetate buffer (0.1 M, pH 4.8), 10 mg of Avicel as the substrate and 1.0 mL of diluted enzyme solution. The reducing sugars released were analyzed via the dinitrosalicylic acid (DNS) assay (Fang et al. 2008; Miller 1959). Xylanase activity was assayed in a 1.0-mL reaction mixture containing 1% (w/v) birch wood xylan, 50 mM acetate buffer (pH 5.0) and appropriately diluted enzyme solutions. Following 30 min incubation at 45 °C, the reducing sugar liberated was measured using the DNS assay. Mannanase activity was measured under similar conditions as xylanase activity, with the exception that 1% locust bean gum (glucomannan) served as the substrate. For these experiments, one unit of enzyme activity was defined as the amount of enzyme required to produce 1 μmol of reducing sugar per minute.

For the measurement of β-glucosidase activity, appropriately diluted enzyme solution and 10 mM p-nitrophenyl-β-D-glucopyranoside were added to 100 mM citrate buffer, and the enzyme reaction mixture was incubated at 45 °C for 10 min. Absorbance at 420 nm was then measured as described by Ghose, 1987 (Ghose and Chemistry 1987). α-Glucosidase activity was measured under similar conditions; with the exception that p-nitrophenyl-α-D- glucopyranoside was used as the substrates. One unit of glucosidase enzyme activity was referred to the amount of enzyme required to release 1 μmol of nitrophenol per minute of reaction. The extracellular lipolytic activity was evaluated according to method described by (Hung et al. 2003). Briefly, the reaction mixture contained 1 mL of 0.05 M phosphate buffer (pH 8), 0.1 mL of enzyme source (supernatant) and 1 mL of 0.013 M p-nitrophenyl palmitate (p-NPP) in ethanol. It was mixed for 5 min at 30 °C. To terminate the reaction, 2 mL of 0.5 M Na_2_CO_3_ was added to the mixture. Then, the mixture was subjected to the centrifugation at 10,000 rpm for 10 min and the absorbance of the mixture was read at 410 nm. One unit (U) of lipase activity was designated as the amount of enzyme required to release 1 µmol/min of p-nitrophenol under the assay conditions. All the activity measurements were performed in triplicate.

### Screw press method

Mechanical pressing is the best way to increase the density of fiber then increase the total heating value. The main parts of this fiber press comprised the control unit (control on temperature and speed), the heaters, and the screw press unit. The maximum rotational speed is 140 rpm because the motor speed is 1400 rpm and the gearbox reduction ratio is 1/10. The final speed is controlled from 1 to 140 rpm by a frequency inverter. The best conditions were used to pressing fiber and give high densities are rotational speed of 30 rpm and a preheating temperature of 60 °C where CTF/COW and tCTF/COW for non-treated and treated mixture, respectively.

### Characterization

Characterizations of structural changes performed by many different instruments included ATR-FTIR spectroscopy (Spectrum Two IR Spectrometer—PerkinElmer, Inc., Shelton, USA). All spectra were obtained by 32 scans and 4 cm^−1^ resolutions in wavenumbers ranging from 4000 to 400 cm^−1^. The surfaces were investigated by a field emission scanning electron microscopy (SEM) Model Quanta 250 FEG (Field Emission Gun) for analysis and mapping, with accelerating voltage 30 kV. TGA scan was carried out using the TGA Q500 device.

### Heating value test

Heating value is a primary parameter availed in the design process of the thermochemical conversion apparatus of fiber and heat sources. The oxygen with a purity higher than 99.5% was used to fill the oxygen bomb (Al Sabagh et al. 2016; Shen et al. 2012). Experimental apparatus, an isothermal oxygen bomb calorimeter (Egyptian Petroleum Research Institute (EPRI) in Egypt) was used. The inner barrel of the calorimeter was filled with deionized water whose temperature was measured to the accuracy of 10–4 Kat intervals of 30 s by using a high accuracy digital thermometer. In addition, the sample and the mixture were pressed by using a laboratory-scale tablet machine. The samples were weighed by using an electronic balance with a minimum sensitivity of ± 0.1 mg.

## Results and discussion

### Citrus trees fibers and cooking oil waste as a substrate for fungal growth

In this study, alkaline pretreatment of CTF was carried out due to its strong pretreatment effect and relatively simple process scheme, also it can remove lignin without degrading carbohydrates, and increases porosity and surface area, thereby enhancing enzymatic hydrolysis (Kim et al. 2016). Fungal strains; *R. oryzae, R. microsporus, A. niger, A. fumigatus, A. flavus* and *A. terreus *were grown on pretreated CTF with and without COW through SSF as shown in Table 1. Results illustrated that *A. flavus* is the best for fungal growth on both alkaline pretreated CTF with and without COW among other fungal strains. The high growth of *A. flavus* may be due to its secrets high amount of cellulases, hemicellulases, lignin peroxidase, laccase as well as lipases which degrade cellulose, hemicellulose, lignin and COW. Therefore, *A. flavus* was selected for further experiments. For knowledge, these enzymes which produced by *A. flavus* were estimated in both alkaline pretreated CTF with and without COW at different pH in the next experiment.

**Table 1:** Fungal growth of different fungal strains on pretreated CTF with and without COW

Fungal strain	Fungal growth	
	Pretreated CTF only	Pretreated CTF + COW
*Rhizopus oryzae*	−	+
*Rhizopus microsporus*	+	+
*Aspergillus niger*	++	++
*Aspergillus fumigatus*	+	++
*Aspergillus flavus*	+++	+++
*Aspergillus terreus*	+	+

+++, ++, + and − mean high, moderate, low and no growth, respectively

### Enzyme evaluation of the extracted culture supernatant

In order to investigate the citrus trees fibers degradation enzymes which directly contribute in the improvement of CTF characteristics, we initially determined the xylinolytic, cellulolytic and ligninolytic enzyme activities in both CTF and it's supplemented with COW culture media. In this regard, the enzymes Avicelase, CMCase, α-glucosidase and β-glucosidase activities were used to evaluate the cellulase activity, and xylanase, mannanase activities were used to evaluate hemicellulase activity, laccase, and lignin peroxidase activities were corresponding to the ligninolytic enzyme activity (Table 2). Also, the lipolytic activity reflects the COW utilization as supplement carbon source and that appeared via degradation process by *A. flavus.*

**Table 2:** Screening of different enzymes resulting from growth of *A. flavus* on CTF

No.	Enzymes (U/gds)	CTF (U/gds)				CTF + COW (U/gds)		
		Extraction of enzymes using different pH values						
		5	7	9	5		7	9
1	CMCase	13.15 ± 0.74	24.62 ± 1.28	14.54 ± 0.53	36.96 ± 0.78		45.73 ± 1.06	48.46 ± 0.57
2	Avicelase	0.846 ± 0.10	0.219 ± 0.02	0.033 ± 0.003	15.13 ± 0.75		9.6 ± 0.78	8.03 ± 0.33
3	Xylanase	50.63 ± 1.31	31.4 ± 2.4	15.3 ± 1.52	34.2 ± 1.63		23.4 ± 2.69	11.96 ± 1.79
4	Mannanase	23.05 ± 1.39	17.03 ± 0.86	12.27 ± 1.51	19.12 ± 1.11		15.43 ± 1.30	12.46 ± 0.74
5	α-Glucosidase	2.30 ± 0.30	4.11 ± 0.73	0.05 ± 0.02	13 ± 1.35		12.06 ± 1.5	5.43 ± 0.57
6	β-Glucosidase	7.13 ± 0.49	7.53 ± 0.44	0.70 ± 0.11	21.4 ± 1.09		25.4 ± 0.53	2.93 ± 0.57
7	Laccase	ND	ND	ND	ND		ND	ND
8	Lignin peroxidase	0.18 ± 0.02	0.35 ± 0.16	0.06 ± 0.016	0.080 ± 0.009		0.63 ± 0.16	0.008 ± 0.002
9	Lipase	3.73 ± 0.26	2.26 ± 0.57	3.1 ± 0.49	18.03 ± 0.97		22.46 ± 1.26	20.83 ± 1.55

*ND* not detected

After incubation time of the *A. flavus* culturing under solid-state fermentation, the enzyme mixture were isolated from the solid cultures using different buffers system (ranging from 5 to 9 for 2 h. at 180 rpm and 30 °C) in order to determine the change of fibers by the enzymatic action. As can be seen in Table 2, the maximum extraction of cellulase enzymes was found at pH. 7, while the highest isolation of hemicellulase enzymes was obtaining at pH. 5. Significantly, enzyme activities in CTF supplemented with COW cultures was considerably higher than in CTF cultures in terms of cellulase enzymes. In contrast, the hemicellulase enzymes were significantly superior under CTF culture medium than in CTF supplemented with COW cultures. The CTF supplemented with COW had a much higher cellulase activities compared with hemicellulase, especially CMCase enzyme activity. It suggests that cellulolytic enzymes were preferably induced during cultivation with CTF supplemented with COW culture medium. Similarly, the ligninolytic enzyme activities were found induced in the COW–citrus trees fibers culture medium in terms of lignin peroxidase. In addition, the maximum lipase production was obtained in the CTF supplemented with COW culture medium (22.46 ± 1.26 U g^−1^), which becomes a greater than tenfold increase in activity if compared to the citrus trees fibers culture medium (2.26 ± 0.57 U g^−1^). In addition, the mannan-degrading enzyme activity of citrus trees fibers supplemented with COW culture was close to that of citrus trees fibers culture, and the extraction of enzyme was found to be active across the different pH buffers.

As shown in Table 2, the CTF supplemented with COW culture significantly induced higher levels of β, α-glucosidase activity than CTF only. This lower activity of β, α-glucosidase implies accumulation of cellobiose, which is a strong inhibitor of cellobiohydrolase and endoglucanase activities during cellulose hydrolysis (Fujii et al. 2009; Sehnem et al. 2006). In fact, our studies demonstrated that the fungal hydrolyzed cellulosic materials more slowly when cultivating with CTF only. However, the supplementations of CTF with COW generally produce higher levels of lipase activity which contribute in the lipid formation leading to higher levels of cellulase enzymes. In this study, the culture supernatant derived from CTF supplemented with COW culture was proved to display a slight decrease of xylan-hydrolyzing activity than that derived from CTF culture and this result might be related to the differences in culture composition. This suggests that the genes related to xylan-hydrolyzing performance of *A. flavus* may have been suppressed, thus resulting in a reduction in xylanase activity (Fujii et al. 2009). Maximum hemicellulases activities are known to improve when cultivated with carbon sources are rich in hemicellulose like rice straw and bagasse (Kogo et al. 2017).

Accordingly, the enhancement of the CTF under fungal enzymes influence along with COW in order to obtaining a highest heating process is a new method could open a promising way in the alternative energy. Interestingly, selection of alternative lower cost agriculture residues to treat by solid-state fermentation plays a prominent role in the green eco-friendly system, since it benefits in two ways. One of them, providing a possibility to the production of several microbial enzymes with an elevated yield and a lower cost in comparison to the most used culture media (de Cassia Pereira et al. 2015; Rodrigues et al. 2017; Singhania et al. 2010). The other way, the fiber characteristics in these alternative substrates would be improved as a result of the synergistic action of microbial enzymes along with the hydrolyzing COW, thus yielding maximum heating capacity yield.

Indeed, the significant activities of the cellulose-hydrolytic enzymes by fungal cells under solid-state condition have been extensively investigated in the previous reports. It is well evident that *Aspergillus* species have been frequently in these studies as a promising candidate to produce cellulases and hemicellulases complexes (Gomes et al. 2016; Santos et al. 2015). Otherwise, the cellulolytic and hemicellulytic enzyme induction was also dependent on the nature of substrate, since sugarcane bagasse (SCB) and wheat bran (WB) was found to be an excellent substrates for xylanase production by *Aspergillus fumigates* M.7.1 with higher activity (1040 U g^−1^) after 6 days (Moretti et al. 2012)**.**

In accordance of our results, lower productivity of β-glucosidase (3 U g^−1^) by *Aspergillus niger* NS-2 strain using Sugarcane Bagasse (SCB) under SSF condition was also reported by (Bansal et al. 2012; Santos et al. 2015). In addition, induction of β-glucosidase, β-xylosidase and xylanase by the *Aspergillus niger* SCBM3 strain using SCB and Wheat Bran (WB) as substrates was studied by (Bajar et al. 2020), they also investigated  the cellulase and xylanase enzyme production by *A*. *heteromorphus* using anaerobically treated distillery spent wash (ADSW) and rice straw (RS) and revealed that the highest exoglucanase, xylanase and endoglucanase enzyme activities under optimum conditions were 6.3 IU/mL, 11.6 IU/mL and 8.1 IU/mL, respectively.

It is noteworthy to mention that, the higher activities of CMCase, Avicelase, α-glucosidase, and β-glucosidase in the presence of COW can be suggested by the increasing of the metabolic activity of *A. flavus,* in which COW exhibited as a growth promoter encourage the biosynthesis of these enzymes. Where, it can be noted a plausible lipolytic activity in the presence of COW, which can contribute in the increased of the fungal metabolic activity (Hashem et al. 2022). On contrast, the presence of COW was found to correlated with the decrease of xylanase and mannanase which may be attributed to the inhibition effect of COW on the production of these enzymes by accumulation of some fatty acids resulted by COW degradation (Abdelraof et al., 2019). In addition, the lower activities of β-glucosidase and α-glucosidase in both culture media may be due to the intracellular formation nature of these enzymes by fungal strains, which was released at the end of incubation period (i.e., stationary phase) by the autolysis of cells as reported previously (Shahriarinour et al. 2011; Umikalsom et al. 1998).

Based on these comparisons, it can be concluded that *A. flavus* evaluated in the present study was good candidate of cellulolytic enzymes, showing a superior enzymes production leading to enhancement of fiber characteristics along with COW generating high heating energy. To the best of our knowledge, this is the first time that this idea has been reported.

### Fibers analysis

The fibers chemical analysis is tabulated in Table 3 which included Klason`s lignin, cellulose, hemicellulose, ash and wax/resin. The fibers analysis contained raw CTF, tCTF and tCTF/COW. The lignin content was affected by the attachment of lignin peroxidases in both fermentation conditions (fiber with and without oil). The fiber analysis confirmed that the lignin content is changed by non-significant values in both treatment conditions. Otherwise, the cellulose as well as hemicellulose content was affected by a significant value where cellulose was decreased in raw CTF and CTF and COW samples by about 12 and 27%, respectively (Abdelraof et al. 2020; Elleboudy et al. 2021). Additionally, hemicellulose contents were decreased by around 41 and 25%, respectively (Hasanin et al. 2019, 2020). These results were in a good agreement with enzyme evaluation of the extracted culture supernatant part. Herein, fibers composition might be affect the heating value.

**Table 3:** Main chemical compositions of CTF, tCTF and tCTF and COW

Components (%)	Klason`s lignin	Cellulose	Hemicellulose	Ash	Wax/resin
CTF	16.8 ± 1.2	67 ± 1.32	7.8 ± 0.84	8.02 ± 0.55	1.01 ± 0.24
tCTF	16.2 ± 1.09	59.3 ± 2.01	4.6 ± 1.09	19 ± 1.89	0.93 ± 0.87
tCTF and COW	17.3 ± 0.98	49.3	5.9 ± 0.98	26 ± 1.22	ND

### Fiber characterization

#### FTIR

**Fig. 1:**
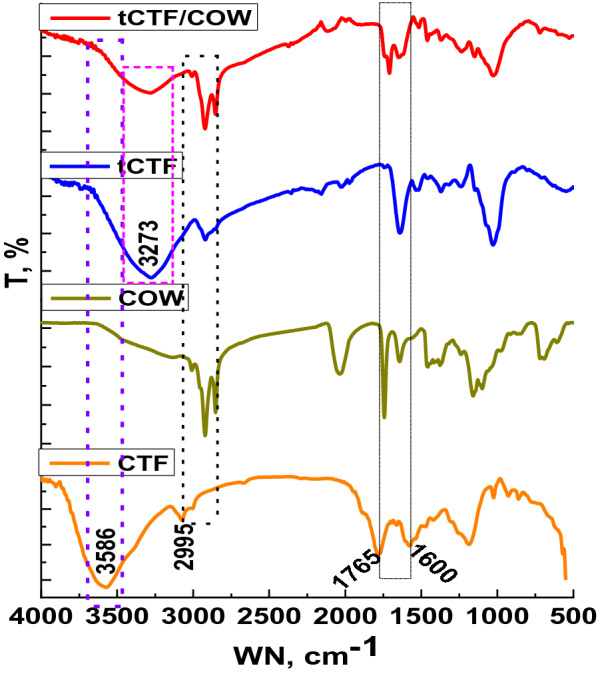
FTIR spectra of raw CTF, tCTF, COW and tCTF/COW

FTIR spectroscopy is the useful technique used to assign the functional groups as well as keep track of changes in main functional groups. Raw CTF, COW, tCTF and tCTF and COW FTIR spectra are presented in Fig. 1. On comparing raw CTF and tCTF, a significant change in raw CTF after fermentation process is observed. The band of OH stretching vibration assigned at 3586 in raw CTF was shifted to low frequency at 3273 cm^−1^. In addition, the band of CH stretching vibration shifted to low frequency at 2912 cm^−1^. This shifting referred to decrease in cellulose ratio as well as hemicellulose. On the other hand, the bands of lignin at around 1589 and 1765 cm^−1^ illustrated a non-significant change. Moreover, the band at 1024 cm-1 which referred to b-glycosidic linkage appeared as sharp band at the same position approximately (Hasanin et al. 2019; Hashem et al. 2020; Youssef et al. 2019). Otherwise, the COW spectrum was assigned the 3012, 2925, 2848, 1737, 1659, 1371 and 979 cm^−1^ which due to OH stretching vibration, =C–C–H, C=C, –CH=CH_2_, respectively (Qiao et al. 2019). In this context, the tCTF/COW spectrum appeared as combination between fiber and oil with main changes in the bands position of fibers as showed in the tCTF. Herein, the tCTF/COW spectrum assigned the significant changes in the COW bands where the main bands were shifted to 3000, 2919 and 1716 cm^−1^. In addition, the band at 979 cm-1 disappeared. These results affirmed that the simplification fibers and oil was observed may be induced the heating value of the tCTF/COW. However, both materials are maintained their microstructure that observed with minor changes.

#### SEM

**Fig. 2:**
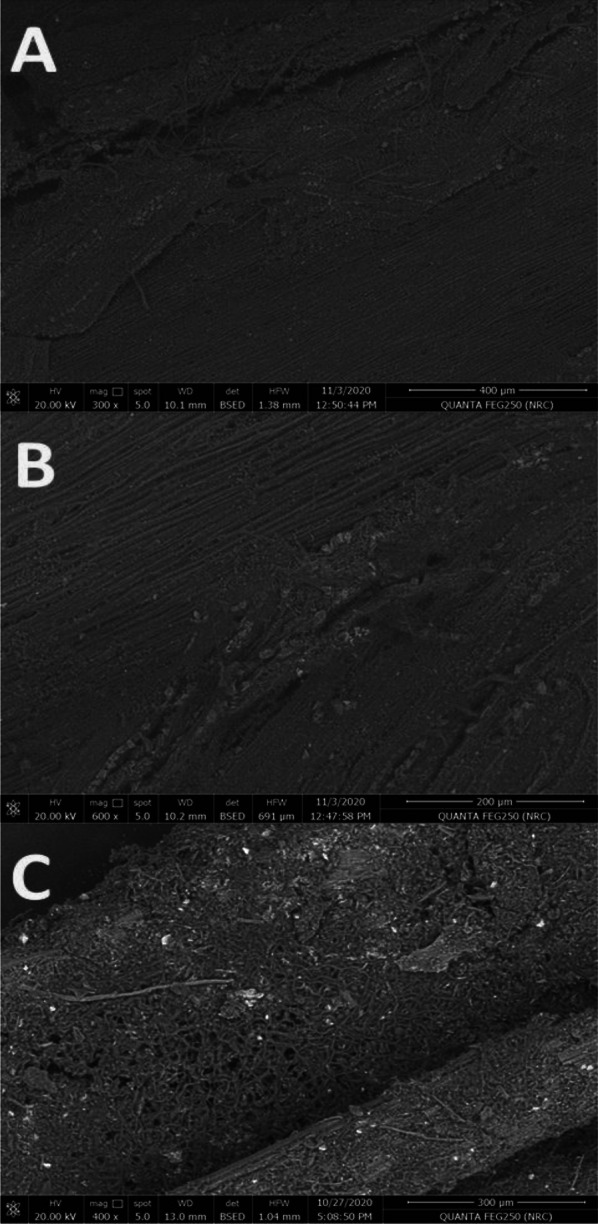
Topography analysis of raw CTF (**A**), tCTF (**B**) and **t**CTF/COW (**C**)

The topography study of the surface morphology of raw CTF, tCTF and CTF/COW is illustrated in Fig. 2. The raw CTF (Fig. 2A), was observed as overlapped fibers collected together as a smooth surface in many points. In addition, the tCTF appeared with less overlapping as well as disappearing of collections and the surface observed as fibers structure morphology. However, the tCTF/COW sample image was illustrated the surface of fibers as pours surface that may be according to the role of oil that penetrated the fibers and induced the fungal growth to attach fibers as well as oil. These observations emphasized that the fungal treatment affected the fibers inter- and intra-molecular structures in present and absent of COW. These observations may be affected the heating value.

### Thermal stability

**Fig. 3:**
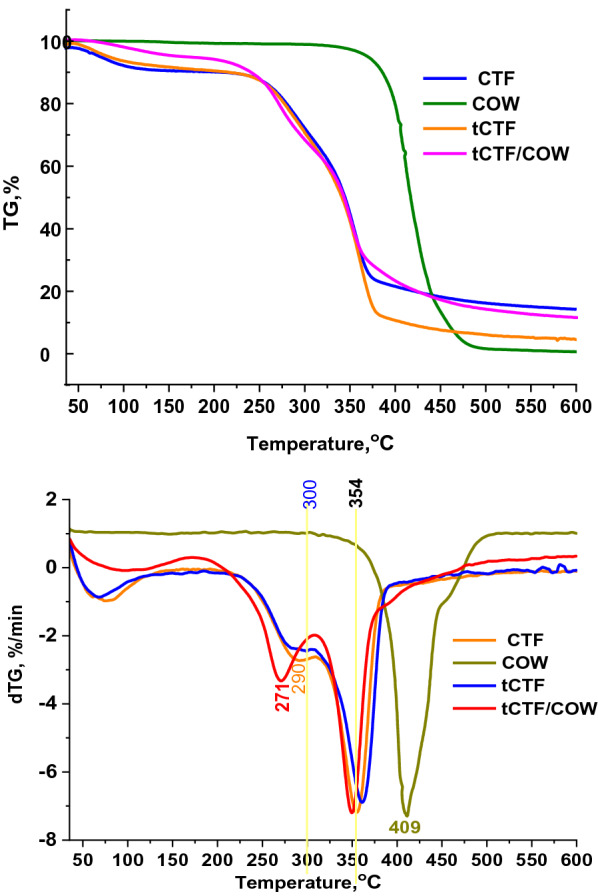
Thermal analysis of samples (TGA (upper) and DTGA (lower))

Figure 3 shows the thermal analysis (TGA and DTGA) of the raw CTF, tCTF and tCTF/COW. The thermal behavior of each sample was related and illustrated the heating value as well as the starting burring attitude. The first decomposition peak was closely with the flashing point of burring. Herein, the COW was recorded the first decomposition peak at 409 °C as a single stage of decomposition. In contrast, all other samples were recorded two decomposition stages. Moreover, the CTF/COW was recorded the lowest decomposition temperature in the first stage of decomposition. Additionally, the second stage of CTF/COW was observed the sharper peak at 350 oC. Otherwise, the raw CTF and tCTF are recorded the first stage decomposition peaks at 290 and 300 °C. These results confirmed that the thermal behavior of untreated CTF and COW was effected significantly after mixing and fermented together. Additionally, these observations affirmed that the fungal enzymes made the coal simple to burn with low flashing point.

### Heating value

**Fig. 4:**
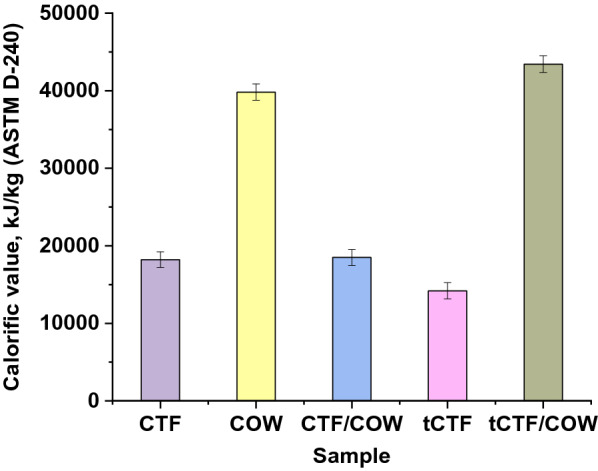
Calorific value of each substrate before and after fungal enzyme treatment

Calorific value is the amount of heat energy present in the fuel, and determined by the complete combustion of a specified quantity at constant pressure and in normal conditions. It is also called calorific power. The measured calorific values of CTF, CTF/COW, tCFF, tCTF/COW, and COW processes were 18,214,18,497,14,200,43,422 and 39,823 kJ/kg, respectively, as shown in Fig. 4. COW is an edible oil that has formerly been used for frying in restaurants and hotels, and no longer be used for similar purposes. In most towns in developing countries including Egypt, waste cooking oil is simply dumped into the environment. COW used to produce solid fuel is environmentally friendly for it recycles waste cooking oil and gives renewable energy with lower pollution. It substitutes some amount of petrochemical oil import and also lowers the cost of waste management. The COW provides alternative energy with a high calorific value to producing solid fuels from biomass for various uses, COW has a high calorific value of 39,823 kJ/kg compared to CFT 18,214 kJ/kg. The screw press was used to increase the density by compressing the fibers in a small volume. This led to an increase in the heating value. COW added to CTF increased the calorific value for the oil fiber mix because of the higher calorific value of oil. *A. flavus* decreased grain size and increased the mixing between the fibers and oil. It led to an increased calorific value of tCTF/COW. The calorific value of fuel determines the availability of heat to produce the power. Therefore, calorific values are important in the choice of alternative fuel for coal for higher performance. The high calorific value of oil and good mixture by *A. flavus*, finally high density by compressed mix using a screw press caused to the higher calorific value of tCTF/COW.

## Conclusion

The current study presented unique eco-friendly method for production of pressed coal based on biowastes. The biowastes (CTF, and CTF/COW) were treated with hydrolytic enzymes produced from *A. flavus* such as xylinolytic, cellulolytic and lipase. Our results revealed that CTF/COW fermented by *A. flavus* in situ provide a significant synergistic induction of considerable amount of hydrolytic enzymes and enhancement the fiber characteristics, where the best pH for enzymes extraction was between 5 and 7. The chemical analysis as well as instrumental analysis of coal and their raw materials emphasized the changes in the chemical composition and chemical bonding which made the pressed coal after fungal enzymatic treatment give the heating value greater than the lone fibers or oil. The calorific values of tCTF/COW was 43,422 kJ/kg, which was higher than CTF (18,214 kJ/kg) and COW (39,823 kJ/kg). Moreover, the screw press increases the density by compressed the CTF in a small volume, COW adding to fibers increased the calorific value. The fungal treatment decreased grain size, and increases the mixing between the CTF and COW, which led to an increased calorific value of tCTF/COW mix.

## Data Availability

The datasets generated during and/or analyzed during the current study are available from the corresponding author on reasonable request.
